# *Epilepsia Open*– December 2023 Announcements

**DOI:** 10.1002/epi4.12868

**Published:** 2023-12-01

ILAE Congresses

13th ILAE School on Pre‐Surgical Evaluation for Epilepsy and Epilepsy Surgery (EPODES Advanced II)

22–26 January 2024

Brno, Czech Republic

4th ILAE School on Neuropsychology in Epilepsy

3–8 March 2024

Lyon, France

https://www.ilae.org/index.cfm?objectid=A1883CAB‐9188‐750C‐76CBC9BE86533EEF

6th ILAE School on EEG in the First Year of Life: From newborn to toddler

25–28 March 2024

Cambridge, UK & Online

https://www.ilae.org/congresses/eeg‐in‐the‐first‐year‐of‐life2

XIII Congreso Latinoamericano de Epilepsia

15–18 June 2024

Santo Domingo, República Dominicana

https://www.ilae.org/index.cfm?objectid=2285A4EC‐0216‐2993‐CA7E9E69B6C6DEC6

15th European Epilepsy Congress

7–11 September 2024

Rome, Italy

https://www.ilae.org/index.cfm?objectid=3ACE9CE6‐B20D‐1E29‐E3E7321523A9DE9F

14th International Summer School for Neuropathology and Epilepsy Surgery

19–22 September 2024

Erlangen, Germany

https://www.ilae.org/index.cfm?objectid=03AFD92A‐BFFB‐8C81‐2DF0C26D46BFD7DE

15th Asian & Oceanian Epilepsy Congress

November 2024

New Delhi, India

https://www.ilae.org/index.cfm?objectid=2349B5B3‐C474‐BD7A‐37160437EE89ECA9

36th International Epilepsy Congress

30 August ‐ 3 September 2025

Lisbon, Portugal

https://www.ilae.org/index.cfm?objectid=1F2693F1‐B1B9‐C59E‐CA592DF4A1C145F3

ILAE Webinars

Definitions and diagnosis of developmental disabilities: Intellectual disability and autism

8 December 2023

https://www.ilae.org/index.cfm?objectid=6A9D5DA1‐08D7‐837B‐AEA6D3309F803790

Apport de la génétique dans la prise en charge des épilepsies

12 December 2023

https://www.ilae.org/index.cfm?objectid=A42BDDB2‐9533‐F293‐BC2346306C480BA7

Nodding Syndrome

15 December 2023

https://www.ilae.org/index.cfm?objectid=A39A2C58‐AA41‐26DF‐2A7D463AFFA78B1A

ILAE‐Asia & Oceania YES Webinar

19 January 2024

https://www.ilae.org/index.cfm?objectid=D50194EE‐EB52‐FCD1‐99EA9FA3DFE80BA4

ILAE e‐Forum: Optimal models of care during transition from childhood to adulthood

26 January 2024

https://www.ilae.org/index.cfm?objectid=F973F05F‐EED3‐B649‐F82882411CCB2602

Living with Epilepsy: Conversation between a patient and a psychologist

26 January 2024

https://www.ilae.org/index.cfm?objectid=8102B8E9‐DD40‐2F03‐3005E9AE18B1FE23

Other Congresses – 2023

AES Annual Meeting 2023

1–5 December 2023

Orlando, Florida, USA

https://www.ilae.org/congresses/aes‐annual‐meeting‐2023

ILAE ASEPA Pediatric EEG Master Class

9 December 2023

Taipei City, Taiwan

https://www.ilae.org/index.cfm?objectid=979B9D70‐9CA7‐60E3‐36AFE8BF2BDD1253

In Search of Lost Time 4

13–15 December 2023

Rome, Italy

https://www.ilae.org/index.cfm?objectid=A65CC476‐E9C5‐694C‐A2A905B382342032

Other Congresses – 2024

Cours Régional ILAE‐EMR d’Epilepsie

15–16 December 2023

Sfax, Tunisie

https://www.ilae.org/index.cfm?objectid=7D0A5C69‐D176‐EF0F‐1DC154AAFD7177EC

1st International Epilepsy Surgery Society Congress

19–21 January 2024

Dubai, UAE

https://www.ilae.org/congresses/1st‐international‐epilepsy‐surgery‐society‐congress

13th ILAE School on Pre‐Surgical Evaluation for Epilepsy and Epilepsy Surgery (EPODES Advanced II)

22–26 January 2024

Brno, Czech Republic

https://www.ilae.org/index.cfm?objectid=898000D2‐C202‐20E3‐9B3E5579B2966043

Alpine EEG Course

25–28 February 2024

Oberstdorf, Germany

https://www.ilae.org/index.cfm?objectid=B622C929‐F957‐9B1C‐30A5AA58AA28A691

17th Latin American Summer School on Epilepsy

16–24 February 2024

São Paulo, Brazil

https://www.ilae.org/index.cfm?objectid=9EA27420‐EC98‐FDF9‐4A7533C79D77F388

4th ILAE School on Neuropsychology in Epilepsy

3–8 March 2024

Lyon, France

https://www.ilae.org/index.cfm?objectid=A1883CAB‐9188‐750C‐76CBC9BE86533EEF

EEG in the First Year of Life

25–28 March 2024

Cambridge, UK & Online

https://www.ilae.org/index.cfm?objectid=93B10B77‐AE3F‐B286‐ECEBBCA9E1DF4A8D

Seventeenth Eilat Conference on New Antiepileptic Drugs and Devices (EILAT XVII)

5–8 May 2024

Madrid, Spain

https://www.ilae.org/index.cfm?objectid=E3F7D4D3‐AB5A‐CB44‐1CA4F4F57EABCDE4

ILAE School on Neuroimaging 2024

15–18 May 2024

Potsdam, Berlin & Online

https://www.ilae.org/index.cfm?objectid=4CD8F446‐9D68‐1D9B‐03255DE5414F1D6F

13th World Congress for NeuroRehabilitation

22–25 May 2024

Vancouver, Canada

https://www.ilae.org/index.cfm?objectid=050F77A5‐F85B‐1B11‐B43BC6F464437983

62. Jahrestagung der Deutschen Gesellschaft für Epileptologie

12–15 June 2024

Offenburg, Germany

https://www.ilae.org/index.cfm?objectid=2129A469‐EDAE‐A29E‐F2502197EE618BF4

10th Congress of the European Academy of Neurology

29 June ‐ 2 July 2024

Helsinki, Finland

https://www.ilae.org/index.cfm?objectid=56EDCEED‐E840‐B337‐916489DBF978B4E8

20th Advanced San Servolo Epilepsy Course

21 July ‐ 1 August 2024

San Servolo (Venice), Italy

https://www.ilae.org/index.cfm?objectid=D09A9B2B‐FDDA‐82F1‐B88B8620749FD6FD

10th Eilat International Educational Course: Pharmacological Treatment of Epilepsy

15–20 September 2024

Jerusalem, Israel

https://www.ilae.org/congresses/10th‐eilat‐international‐educational‐course‐pharmacological‐treatment‐of‐epilepsy

